# In Vitro and In Silico Kinetic Studies of Patented 1,7-diEthyl and 1,7-diMethyl Aminoalkanol Derivatives as New Inhibitors of Acetylcholinesterase

**DOI:** 10.3390/ijms23010270

**Published:** 2021-12-27

**Authors:** Błażej Grodner, Mariola Napiórkowska, Dariusz Maciej Pisklak

**Affiliations:** 1 Chair and Department of Biochemistry and Pharmacogenomics, Medical University of Warsaw, 1 Banacha Street, 02-097 Warsaw, Poland; 2 Department of Biochemistry, Medical University of Warsaw, 1 Banacha Street, 02-097 Warsaw, Poland; mariola.napiorkowska@wum.edu.pl; 3 Department of Physical Chemistry, Medical University of Warsaw, 1 Banacha Street, 02-097 Warsaw, Poland; dpisklak@wum.edu.pl

**Keywords:** acetylcholinesterase inhibitors, Alzheimer’s disease, aminoalkanol derivatives, anticancer drugs

## Abstract

Two aminoalkanol derivatives of 1,7-diEthyl-8,9-diphenyl-4azatricyclo (5.2.1.02.6) dec-8-ene-3,5,10-trione and two derivatives of 1,7-diMethyl-8,9-diphenyl-4-azatricyclo (5.2.1.02.6) dec-8-ene-3,5,10-trione were evaluated in vitro for their inhibition efficacy of acetylcholinesterase. The Km, Vmax, slope angles of Lineweaver–Burk plots, Ki and IC_50_ values showed that all four aminoalkanol derivatives are competitive inhibitors of acetylcholinesterase whose inhibitory potency depends, to a varying extent, on the nature of the four different substituents present in the main compound structure. Studies have shown that the most potent acetylcholinesterase inhibitors are derivatives containing isopropylamine and/or methyl substituents in their structure. In contrast, dimethylamine and/or ethyl substituents seem to have a weaker, albeit visible, effect on the inhibitory potency of acetylcholinesterase. Additionally, docking studies suggest that studied compounds binds with the peripheral anionic site and not enter into the catalytic pocket due to the presence of the sterically extended substituent.

## 1. Introduction

**Figure 1:**
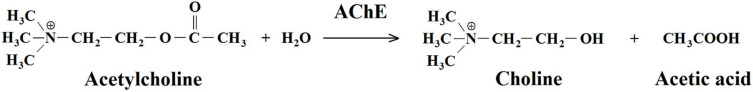
Scheme of acetylcholine decomposition reaction catalyzed by acetylcholinesterase (AChE).

Acetylcholinesterase (AChE; E.C. 3.1.1.7) belongs to the group of hydrolases. The most important function of AChE is the hydrolysis of an important neurotransmitter—acetylcholine—to choline and acetic acid, according to the scheme shown in Figure 1.

Acetylcholine (ACh) is one of the most important neurotransmitters because it is present both in the peripheral nervous system, in the central nervous system and in the autonomic nervous system [1,2,3]. It performs many different functions depending on the place of its operation. The basic structures that interact directly with ACh are nicotinic receptors found in the autonomic ganglia, motor nerve endings and the central nervous system, and the muscarinic receptors found in parasympathetic nerve endings [4,5]. ACh’s main action is to stimulate skeletal muscle contraction. However, the condition for an effective and precise muscle contraction, leading to the intended movement, is the proper secretion of acetylcholine (ACh) in the motor nerve endings and its rapid inactivation. For the latter process to run smoothly, an appropriate enzyme with a specific activity is needed. As mentioned at the beginning, the main enzyme responsible for the breakdown and maintenance of adequate levels of acetylcholine is AChE. ACh also causes blood vessels to widen, which lowers blood pressure. At the same time, it reduces the heart rate and the strength of its contraction. It also causes contractions of the smooth muscles of the bronchi, intestines (thus stimulating their peristalsis) and the urinary bladder. In addition, it causes constriction of the pupils and stimulates the secretory functions of the glands. It is also credited with activating cognitive and memory processes [6].

Certain compounds, such as, for example, organophosphate and carbamate compounds or war gases (sarin, soman, tabun) [7], being irreversible AChE inhibitors, cause a significant elevation of ACh levels. The first symptoms that appear as a result of these inhibitors are constriction of the pupils, followed by pain in the eyeball, bronchospasm with coughing and shortness of breath, chest pain and drooling. Other symptoms include vomiting, abdominal pain with diarrhea, involuntary passing of urine and stools, muscle tremors, followed by convulsions and muscle paralysis. In the final stage, the victim of poisoning usually falls into a coma and suffocates during it. Paralysis of the respiratory muscles and the heart muscle is usually fatal [8]. Conversely, many reversible AChE inhibitors are used in medicine as drugs. Examples include Donepezil, Rivastigmine and Galantamine, which by inhibiting the activity of acetylcholinesterase, simultaneously inhibit the breakdown of ACh in the brain and this in turn leads to an increase in the concentration of acetylcholine and to an increase in communication between nerve cells [9,10,11].

If it were not for acetylcholine, our memory would not function properly and we would have trouble maintaining attention and processing information. Therefore, the deficiency of this substance is often associated with Alzheimer’s disease. Research has shown that people suffering from this form of senile dementia have 73% lower concentration of acetylcholine in the body than healthy people with an average amount of this compound [12].

Too little acetylcholine in the body stops the activation of nerve impulses and reduces the strength of muscle contractions, which can lead to their paralysis. The lack of this component (acetylcholine) also affects the development of myasthenia gravis, i.e., an autoimmune muscle disease. Then, there is general fatigue, drooping eyelids, swallowing becomes difficult, and problems with breathing and double vision appear [13].

The decrease in ACh levels in specific areas of the brain is much greater during wakefulness or NREM sleep than during REM sleep, indicating that ACh exerts a significant influence on the induction (formation) of REM sleep [14,15,16]. Thus, acetylcholine deficiency can cause sleep problems, as it can lead to the disappearance of the so-called REM phase or, as a consequence, to its complete absence, which during this stage, dreams appear.

Regulation of AChE activity is therefore of great importance because by influencing the level/concentration of ACh in synaptic clefts, it causes, depending on the degree of its activity, excessive stimulation or inhibition of the activity of appropriate (nicotinic and muscarinic) receptors, which, as mentioned earlier, consequently leads to changes in many functions of the organism [4,5,6].

In the regulation of acetylcholinesterase, compounds of the nature of activators and inhibitors are used. The AChE activating compounds and the compounds accelerating the decomposition of ACh are substances belonging to various chemical groups. Some of them are simple ions such as Mg^2+^, Ca^2+^, Mn^2+^, and Na^+^ [17]; others are more complex compounds [18,19,20]. A common feature of all activators is the activation of acetylcholinesterase leading to excessive decomposition of acetylcholine, the consequences of which are symptoms of acetylcholine deficiency, constipation, gastroparesis, memory problems, difficulty with word recall when speaking, learning difficulties, dry mouth, dry eyes, orthostatic hypotension, low muscle tone, depressed mood, fast heart rate, chronic inflammation, emotional instability and many other symptoms previously described [21,22,23,24,25,26,27,28,29,30].

In contrast, a feature of AChE inhibitors is the reduction of AChE activity, which in turn causes a reduced degradation of ACh and thus an increase in its concentration in the synaptic cleft and excessive stimulation of cholinergic receptors. This in turn leads to many of the abnormalities of many different bodily functions previously mentioned and ultimately death from respiratory failure from paralysis of the respiratory muscles [31]. The compounds of the type of AChE inhibitors are the aforementioned drugs but also a number of other substances, such as analogs of 9-amino-1,2,3,4-tetrahydroacridine [32].

Recently, there have also appeared articles describing the synthesis of new AChE inhibitors, such as hydroxybenzoic acid amides [33] and quinoxaline derivatives [34]. Another work describes the synthesis of ammonium and betaine derivatives, the potency of which is comparable to the potency of pyridostigmine, but the toxicity of ammonium and betaine derivatives is lower [35].

As we can see, the appropriate ACh level is responsible for the proper course of many physiological processes.

It is also well known that on the maintenance of an adequate level of ACh in various structures of the body has the influence of many factors. One of them is the appropriate activity of AChE. The second one includes all kinds of chemical substances.

Beside the enzymatic activity, AChE is postulated to pose also as non-enzymatic activity, having an influence on many physiological pathways, including as cell adhesion, neurite outgrowth and amyloid fibril formation. The peripheral anionic site (PAS), located around the external region of AChE narrow gorge, serves as the secondary binding site of ACh and quaternary ligands without enzymatic activity and are accountable for some of non-catalytic activities. For example, one of the hypotheses regarding amyloid β (Aβ) in Alzheimer’s disease (AD) suggests that Aβ deposition is an important pathogenic marker of the onset and progression of AD. Study from Inestrosa et al. [36] revealed AChE as a molecular chaperone, which accelerates Aβ assembling into oligomers and fibrils in amyloidosis via peripheral anionic site (PAS). Thus, AChE inhibitors blocking peripheral sites might act as disease-modifying agents delaying amyloid plaque formation. This is why it was crucial to predict the possible interaction site of studied compounds and estimate if they would preferentially interact with the active site of an enzyme or bind to the PAS of AChE.

**Figure 2:**
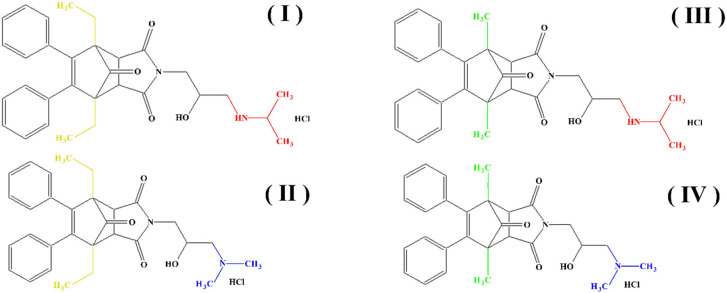
Structures of aminoalkanol derivatives (**I**), (**II**), (**III**) and (**IV**).

Acetylcholinesterase plays an extremely important role in maintaining an appropriate level of acetylcholine in many areas of the body and has an influence on many biochemical pathways. Therefore, in this work, we decided to investigate the inhibition effects of aminoalkanol derivatives of 1,7-diEthyl-8,9-diphenyl-4azatricyclo [5.2.1.02,6] dec-8-ene-3,5,10-trione (I) and (II) and derivatives of 1,7-diMethyl-8,9-diphenyl-4-azatricyclo [5.2.1.02.6] dec-8-ene-3,5,10-trione (III) and (IV) on acetylcholinesterase. These compounds (Figure 2) were evaluated in vitro for their inhibition efficacy of acetylcholinesterase.

The synthesis, chemical characterization, and anticancer activity of the aforementioned compounds (I), (II), (III) and (IV) were described previously in a patent application [37]. Toxicity of the studied compounds for chronic myelogenous leukemia cells, K562 and HeLa cells was in the range 400–100 μM [37].

These compounds were also investigated in our previous works on the possibility of their determination [38,39,40,41] and their inhibitory effect on alkaline phosphatase [42] and prostate acid phosphatase [43]. Interesting results described in our previous works [42,43] prompted us to conduct research on the influence of the mentioned aminoalkanol derivatives with potential anticancer activity on the enzymatic activity of acetylcholinesterase—an enzyme extremely important in our body.

In this study, we used, developed by us, the capillary electrophoresis (CE) method to determine the kinetic and inhibition parameters of acetylcholinesterase in the presence of (I), (II), (III) and (IV) inhibitors.

## 2. Results

In this study, the inhibition of enzymatic activity was analyzed in order to determine AChE inhibition by aminoalkanol derivatives.

### 2.1. Experimental

**Figure 3:**
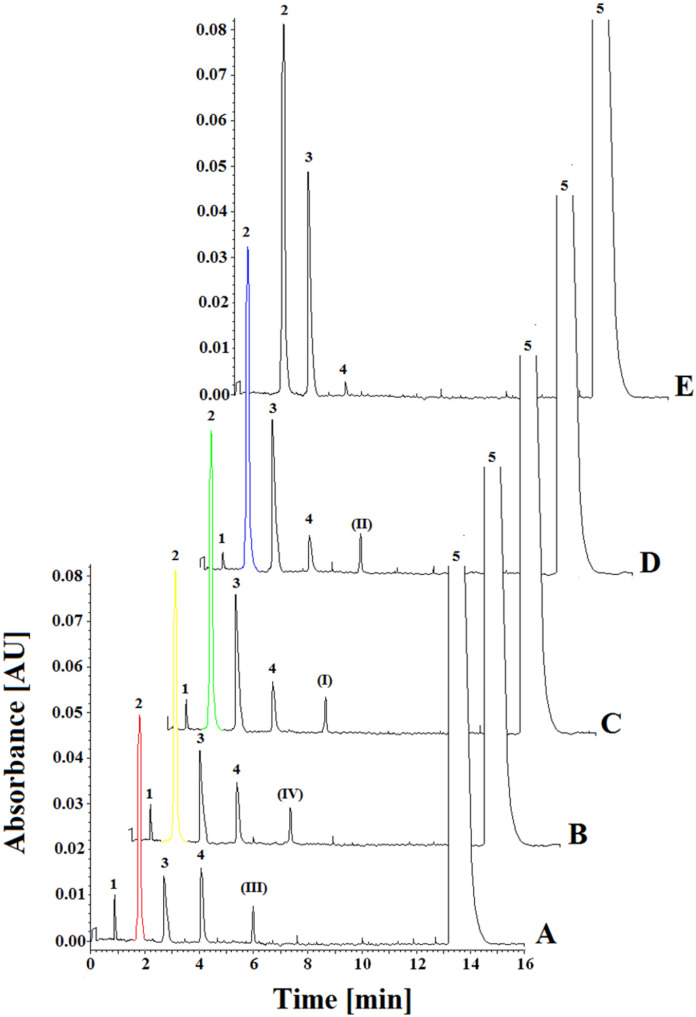
Representative electrophoregrams of investigated compounds in the presence of (**I**), (**II**), (**III**) and (**IV**) AChE inhibitors at the concentration of 0.08 mM and (1) 1.44 mM of acetylthiocholine (ACth), (2) 4,4-dithio-bis-acid nitrobenzoic (DBAN), (3) 5-thio-2-nitrobenzoic acid (NTB), (4) 5,5′-dithio-bis-2-nitrobenzoic acid (DTNB), (5) acetylcholinesterase (AChE). (**A**): (2) 0.41 mM of 4,4-dithio-bis-acid nitrobenzoic (DBAN) in the presence of inhibitor (**III**). (**B**): (2) 0.48 mM of 4,4-dithio-bis-acid nitrobenzoic (DBAN) in the presence of inhibitor (**IV**). (**C**): (2) 0.52 mM of 4,4-dithio-bis-acid nitrobenzoic (DBAN) in the presence of inhibitor (**I**). (**D**): (2) 0.58 mM of 4,4-dithio-bis-acid nitrobenzoic (DBAN) in the presence of inhibitor (**II**). (**E**): (2) 0.66 mM of 4,4-dithio-bis-acid nitrobenzoic (DBAN) without inhibitors.

Capillary electrophoresis method (CE) enabled us to carry out selective monitoring of acetylcholinesterase human (AChE), acetylthiocholine iodide (ACth), 5,5-dithiobis (2-nitrobenzoic) acid (DTNB), 4,4-dithio-bis-acid nitrobenzoic (DBAN), 5-thio-2-nitrobenzoic acid (NTB), and compounds (I), (II), (III) and (IV) (Figure 3).

**Figure 4:**
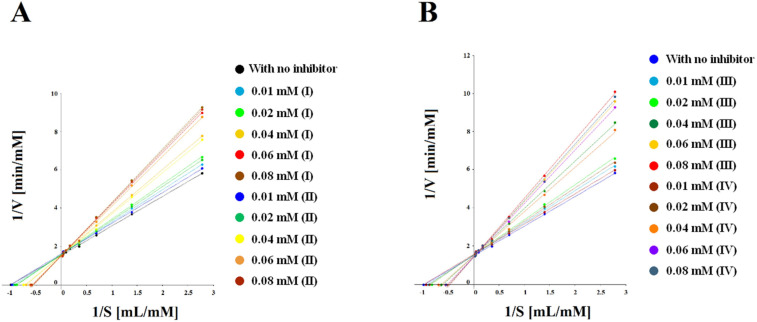
Lineweaver–Burk plots for systems with inhibitors (**I**) and (**II**) (**A**) and (**III**) and (**IV**) (**B**) at concentrations of 0.01, 0.02, 0.04, 0.06 and 0.08 mM.

In this work, we used the combined Ellman’s method with the capillary electrophoresis method developed by us for the determination of 4,4-dithio-bis-acid nitrobenzoic (DBAN) (main enzyme reaction product) in the presence of acetylthiocholine (ACth), 5-thio-2-nitrobenzoic acid (NTB), 5,5′-dithio-bis-2-nitrobenzoic acid (DTNB), acetylcholinesterase (AChE) and inhibitors (I), (II), (III) and (IV) of acetylcholinesterase. For the development of this method, we optimized the buffer pH, separating background electrolyte (BGE) concentration, wavelength, temperature, and voltage. The separation of all compounds was investigated at three pH values (7.0, 7.5, and 8.0), three temperatures (20, 25, and 30 °C), four voltages (5, 10, 15, and 20 kV), three concentrations of separating BGE (20, 30, and 50 mM), and four wavelengths (280, 300, 412, and 420 nm). All these separation systems were tested to achieve the best separation parameters, resolution, and analysis time for all components in the reaction mixture. The best separation of 4,4-dithio-bis-acid nitrobenzoic (DBAN), acetylthiocholine (ACth), 5-thio-2-nitrobenzoic acid (NTB), 5,5′-dithio-bis-2-nitrobenzoic acid (DTNB), acetylcholinesterase (AChE) and compounds (I), (II), (III), (IV) was obtained with 100 mM phosphate buffer (pH 7.5). The measurements were taken at 412 nm with a fused-silica capillary (effective length: 20 cm, diameter: 50 μm). The capillary temperature was 20 °C, which allowed the best resolution to be achieved. Separation was carried out at 15 kV. Each sample was added to the capillary under hydrodynamic injection. Average detection times for DBAN, ACth, NTB, DTNB, AChE, and compounds (I), (II), (III), (IV) were 0.85, 1.79, 2.75, 4.10, 6.00 and 13.25 min, respectively (Figure 2). The constructed curves were linear over the concentration range of 0.36–46.00 mM used for ACth. The basic enzymatic activity was investigated using nine concentrations of reaction substrate (ACth) (0.36, 0.72, 1.44, 2.88, 5.75, 11.50, 23.00, 34.50, and 46.00 mM) in the presence of the enzyme (AChE). The effect of inhibition of compounds (I), (II), (III) and (IV) was determined by an enzymatic kinetics study in a system containing successively increasing concentrations of the substrate at a constant concentration (0.08 mM) of inhibitor (I), (II), (III) and (IV). The correlation coefficient for ACth in the absence of inhibitors was found to be 0.998, and the slope of the curve was 1.546. At the concentration of 0.08 mM for compounds (I), (II), (III) and (IV), the correlation coefficients were determined at 0.997–0.998, and the curves slopes at 2.736–4.019 for compounds (I), (II), (III) and (IV), based on the inhibition type and inhibitory strength (Table 1, Figure 4).

The statistical power for each dataset and each value of the kinetic parameter was achieved by measuring six times each value obtained for the nine concentrations of substrates and reaction products in the system without inhibitors and for four systems in the presence of 0.08 mM inhibitor (I), (II), (III) and (IV). After exceeding the value of 0.08 mM, there was no further increase in inhibition; therefore, the value of 0.08 mM was considered the cutoff value for both inhibitors.

The inhibitory effects of compounds (I), (II), (III) and (IV) on AChE are shown in the form of electrophoregrams in Figure 3, with the detailed data presented in Table S1 in the Supplementary Materials Section.

### 2.2. Analysis

The first stage of the work was to conduct kinetic studies in order to obtain information on the AChE inhibition mechanism with the use of (I), (II) and (III), (IV) aminoalkanol derivatives. Based on the inhibition data obtained in the steady-state state, Lineweaver–Burk plots (Figure 4) were prepared showing the reciprocal relationship of the reaction substrate concentration (acetylthiocholine) to the reciprocal of the reaction rate.

Plots of straight lines with different slope angles (Km/Vmax) obtained for the five inhibitor concentrations (I), (II) and (III), (IV) intersected at one point on the y axis corresponding to the reciprocal value of the reaction rate (Vmax). Such a system is suggested by a reversible competitive AChE inhibition. The calculated Km and Vmax values for systems with different concentrations of compounds (I), (II) and (III), (IV) also indicate a competitive type of inhibition (Table 2).

The next goal of the work was to study the inhibitory strength and the binding strength of individual inhibitors (I), (II) and (III), (IV) to the active center of AChE. For this purpose, based on the obtained values of kinetic parameters, the values of Michaelis-Menten constants (Km), maximum rates (Vmax), inhibition constants (Ki) and half maximal inhibitory concentration (IC_50_) were calculated for five different concentrations of inhibitors (I), (II) and (III), (IV) (Table 2).

The results obtained in this way indicated a competitive type of inhibition, as evidenced by the variable (increasing) values of Km (from 1.07 to 1.83 mM/mL) for compound (I), (from 1.05 to 1.79 mM/mL) for compound (II), (from 1.09 to 1.90 mM/mL) for compound (III) and (from 1.08 to 1.80 mM/mL) for compound (IV) and common, similar Vmax values (oscillating in the range from 0.62 ± 0.03 to 0.65 ± 0.02 mM/min) for four inhibitors (Table 2). Initially, based on the differences in the Km values between individual compounds, it can be shown that the strongest competitive inhibitor of AChE is compound (III), as evidenced by the highest Km values for the aminoalkanol derivative (III) (from 1.09 to 1.90 mM/mL). Another, weaker inhibitor was derivative (IV) (Km from 1.08 to 1.80 mM/mL), then derivative (I) (Km from 1.07 to 1.83 mM/mL). The weakest competitive inhibitor of AChE was derivative (II) (Km from 1.05 to 1.79 mM/mL).

The next value taken into account to determine the inhibition force were the angles of the Lineweaver–Burk straight lines, the values of which increased with the increase of the concentration of inhibitors (I), (II) and (III), (IV) to the concentration limit value of 0.08 mM above which a further increase in inhibition was not observed. The slope angles were the largest for derivative (III) (from 60.01° to 71.50°), smaller for derivative (IV) (from 59.58° to 70.40°), then derivative (I) (from 59.52° to 70.32°) and the smallest for derivative (II) (from 59.46° to 70.02°) (Table 2). Comparisons of Lineweaver–Burk angles for five inhibitor concentrations of (I), (II) and (III), (IV) compounds, also suggested that derivative (III) was the strongest competitive inhibitor of AChE and derivative (II) was the weakest (Table 2, Figure 4).

Then, the inhibition constants (Ki) were calculated for the inhibitors (I), (II) and (III), (IV) with sequentially increasing concentrations (Table 2).

Ki values obtained at five concentrations (0.01 mM, 0.02 mM, 0.04 mM, 0.06 mM and 0.08 mM) of inhibitor (III) compared with Ki values for inhibitor (IV), for inhibitor (I) and for inhibitor (II) (Table 2) indicate the strongest binding of inhibitor (III) to the AChE active site compared to inhibitors (IV), (I) and (II). It can therefore be concluded that compound (III) is the strongest AChE inhibitor, while compounds (IV) and (I) were weaker inhibitors and the weakest AChE inhibitor was compound (II).

**Figure 5:**
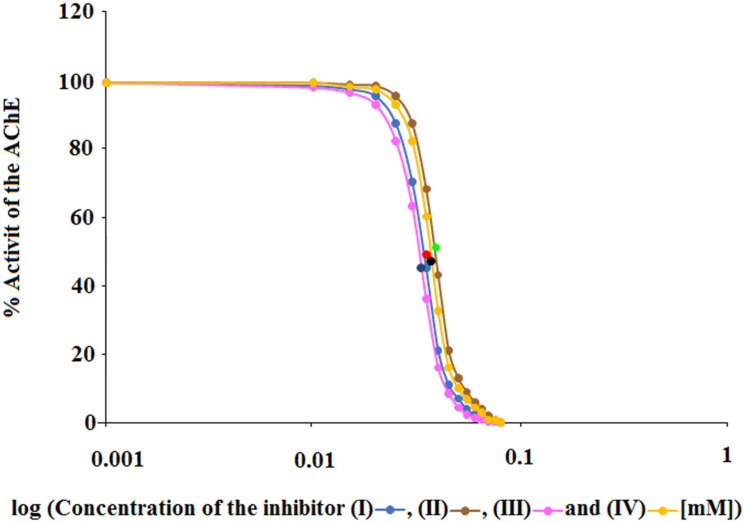
Graphical determination of IC_50_ for (**I**), (**II**), (**III**) and (**IV**) acetylcholinesterase inhibitors.

In order to obtain information regarding the amount of individual compounds (I), (II), (III) and (IV) needed to inhibit AChE by 50% and thus obtain additional values characterizing the potency of compounds (I), (II), (III) and (IV) in inhibiting AChE, the half maximal inhibitory concentration (IC_50_) was calculated (Table 2, Figure 5).

Based on a comparison of the IC_50_ values for compound (III) (0.033 ± 0.001 mM), compound (IV) (0.035 ± 0.001 mM), compound (I) (0.037 ± 0.001 mM) and compound (II) (0.039 ± 0.001 mM) it can be seen that compound (III) causes the inhibitory effect of AChE at a lower concentration than compounds (IV), (I) and (II). Thus, it can be said that also in this case compound (III) turned out to be the strongest AChE inhibitor, while inhibitors (IV) and (I) showed weaker inhibition. The weakest AChE inhibitor was compound (II).

Summarizing, it can be said that all the obtained results indicate a competitive type of AChE inhibition by aminoalkanol (I), (II), (III) and (IV) derivatives. Moreover, the research carried out indicated that the inhibitory potency and affinity strength of all four derivatives depend on the type of four substituents (isopropylamine, dimethylamine, ethyl and methyl) present in the main chemical molecule of the compounds (I), (II), (III) and (IV) (Table 3).

Taking into account the known spatial structure of acetylcholinesterase and the structure of competing inhibitor molecules, we also analyzed the possibility of their interaction with the enzyme’s active site. The active site of AChE is known to be composed of two sites: the catalytic site and the peripheral anionic site (PAS). The AChE catalytic site consists of two sub-sites: a catalytic site composed of a triad (Ser 203, Glu 334, His447) and an “anionic” site that binds the positively charged part of the ligand with the amino acid Tyr 86 of an enzyme. PAS lies at the entrance to the active site consists of 5 residues (Tyr 72, Asp 74, Tyr 124, Trp 286, Tyr 341) clustered around the entrance to the gorge of the active site.

In order to estimate the potential bioactive conformation of the compounds and possible interactions stabilizing the protein-ligand interaction, molecular docking methods were used. Basic question was how the studied molecules can bind with the active site of AChE and if they, by the presence of sterically expanded imide moiety, can enter to the center of active site and interact with the catalytic triad. For docking studies two crystal structures of human Acetylcholinesterase enzyme were selected, one structure was crystallized with the model inhibitor donepezil (6o4w), which binds with both sites, and the second one crystallized with 9-aminoacridine (6o4x), which binds selectively with PAS.

Compounds I, II, III, IV as well as the model compound, donepezil, were all docked in the crystallographic structure AChE active site using the Autodock Vina program.

The obtained values of the scoring function for all tested compounds were significantly lower compared to donepezil for which the estimated binding was estimated at −11.1 kcal/mol. The highest values were obtained for compound IV and III, estimated at −9.2 kcal/mol and −8.9 kcal/mol, respectively. Slightly lower results were obtained for compounds I and II, for which the predicted values estimated to be −8.1 kcal/mol and −8.5 kcal/mol, respectively. This suggested that the ethyl substituent lower the affinity for the AchE enzyme in comparison to the methyl group, which was consistent with the experimental data.

**Figure 6:**
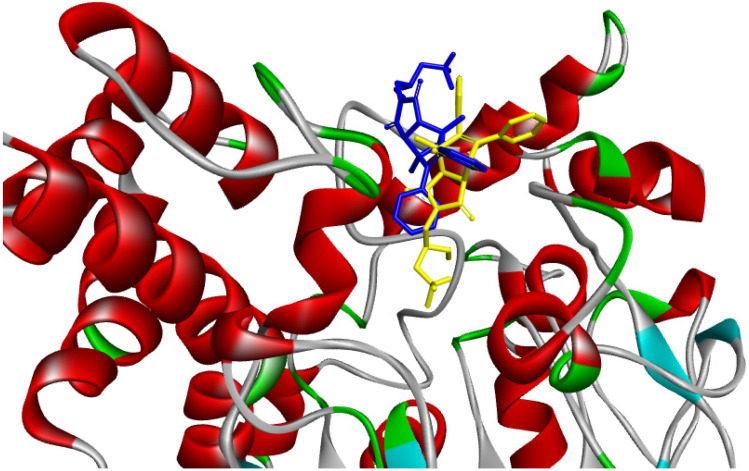
Two binding modes of compound IV in peripheral active site of AChE enzyme (a—yellow b—blue).

Analysis of bioactive conformations showed that studied compounds by the presence of spatially expanded imide substituent cannot interact directly with the catalytic site of the enzyme. Through the slight diversity of structures, the bioactive conformations and intramolecular interactions in the ligand-enzyme complex is similar among all the compounds. Docking studies showed that they all bind with the peripheral anionic site (PAS) at the entrance to the active site gorge and can adopt two possible binding modes (Figure 6).

**Figure 7:**
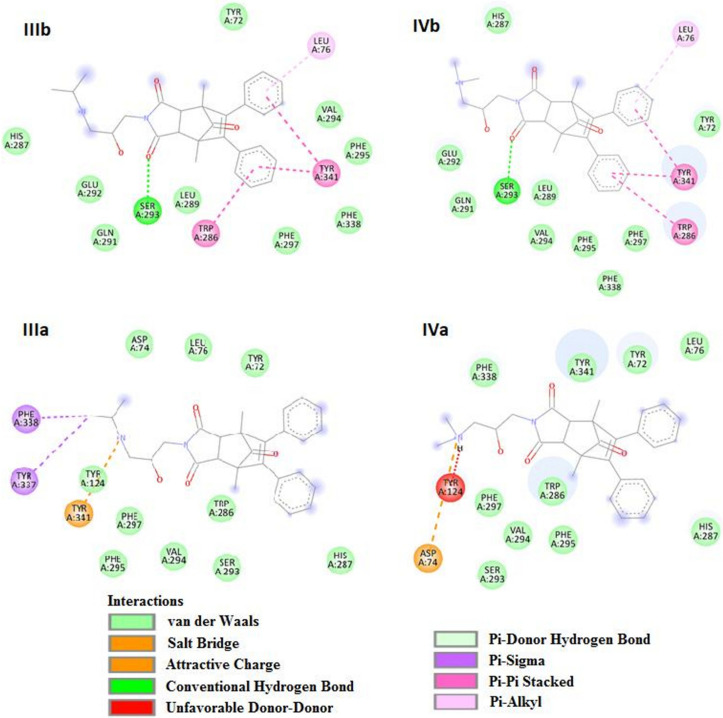
Structure of the active site of AChE and potential types of interactions of two binding modes (**a**,**b**) between the competitive inhibitors (**III)** and (**IV**) and amino acids in the PAS of the enzyme.

In one of them (a), the charged aminoalkanol chain enter the gorge, but cannot reach the active site of an enzyme and the hydrophobic aromatic rings are exposed to the environment. In such a case for compounds II and IV containing N, N-dimethyl moiety bioactive conformation is stabilized by the electrostatic interaction with Asp 74 but simultaneously destabilized by the unfavorable donor–donor interaction with Tyr 124. For compounds I and III with N-isopropyl group, binding with the enzyme is mostly stabilized by interaction with aromatic amino acids Tyr 341, Tyr 337, and Phe 338. In the other binding mode (b), the aromatic hydrophobic moiety interact by π–π interaction with aromatic amino acids forming PAS (Tyr 341, Trp 286) and hydrophobic interaction with Leu 76. Additionally, hydrogen bonds between the carbonyl group of imide part and Ser 293 stabilize the interaction. In this binding mode, the charged amine group is exposed to the environment. Obtained binding interactions with the enzyme are depicted in Figure 7.

## 3. Discussion

Comparing the inhibition potency (IC_50_) of aminoalkanol derivatives (I), (II), (III) and (IV) with model compounds such as Physostigmine, Donepezil, Rivastigmine and Donepezil derivatives [44], it can be seen clearly that they rank in fourth place out of all 14 acetylcholinesterase inhibitors [44].

Inhibition potency of Donepezil (one of the strongest AChE inhibitors) is 0.027 μM, which makes aminoalkanol derivatives (I) (IC_50_ = 37 μM), (II) (IC_50_ = 39 μM), (III) (IC_50_ = 33 μM) and (IV) (IC_50_ = 35 μM)) 1370, 1444, 1222 and 1296 times less AChE inhibitors than Donepezil, respectively. In the case of S-I 26 of the Donepezil derivative (IC_50_ = 14 μM), aminoalkanol derivatives are only 2.6, 2.8, 2.4 and 2.5 times weaker inhibitors of AChE, respectively, compared to S-I 26 of Donepezil derivative [44].

Comparing the inhibition potency of aminoalkanol derivatives (I), (II), (III) and (IV) with Physostignmine, for which IC_50_ = 0.18 μM, it can be seen that in this case aminoalkanol derivatives (I), (II), (III) and (IV) are only 206, 217, 183 and 194 times weaker inhibitors of AChE compared to Physostigmine [44], respectively.

The situation is completely different when we compare the inhibition potency of aminoalkanol derivatives (I), (II), (III) and (IV) with Rivastigmine, for which IC_50_ = 71 μM. In this case, aminoalkanol derivatives (I), (II), (III) and (IV) are respectively 1.9, 1.8, 2.2 and 2.0 times more potent inhibitors of AChE compared to Rivastigmine [44].

Compared to the other Donepezil derivatives [40], the inhibition potency of aminoalkanol derivatives (I), (II), (III) and (IV) is much higher, which means that aminoalkanol derivatives (I), (II), (III) and (IV) are much more potent (from 3.6 to 29.8) inhibitors of AChE compared to Donepezil derivatives [45].

In this study, we also compared the values of the affinity strength (Ki) of aminoalkanol derivatives (I), (II), (III) and (IV) with the strength of the affinity of Donepezil, Physostigmine and Rivastigmine for AChE.

Depending on the form and place of AChE occurrence in the three main brain structures, i.e., in cortex, hippocampus and striatum, the Ki values for Donepezil range from 1.4 × 10^−1^ M to 5.6 × 10^−6^ M, for Physostigmine from 3 × 10^−8^ M to 9.7 × 10^−7^ M, and for Rivastigmine from 1.5 × 10^−5^ M to 1.5 × 10^−3^ M [46].

Depending on the structure of the compounds, the Ki values for aminoalkanol derivatives (I), (II), (III) and (IV) are 1.35 × 10^−3^ M, 1.42 × 10^−3^ M, 1.11 × 10^−3^ M and 1.22 × 10^−3^ M respectively.

Thus, when comparing the Ki values for Donepezil, Physostigmine and Rivastigmine with the Ki values for aminoalkanol derivatives (I), (II), (III) and (IV), it can be concluded that the affinity strength of aminoalkanol derivatives to AChE is from 7.93 × 10^6^ to 2.53 × 10^2^ times weaker than Donepezil, from 3.7 × 10^4^ to 1.46 × 10^3^ times weaker than Physostigmine but comparable to the Ki range of Rivastigmine (from 1.5 × 10^−5^ M to 1.5 × 10^−3^ M).

Looking closer at the Ki values for Rivastigmine, it can be concluded that the affinity strength of aminoalkanol derivatives (I), (II), (III) and (IV) is respectively 1.11, 1.06, 1.35 and 1.23 times greater than the affinity strength of Rivastigmine to the AChE occurring in the cortex.

The research results showed that isopropylamine and dimethylamine substituents exerted a much greater AChE inhibitory effect than diethyl and dimethyl derivatives, while isopropylamine derivatives showed a stronger inhibitory effect than dimethylamine derivatives and they were the strongest AChE inhibitors. Dimethylamine derivatives were less potent inhibitors. Ethyl and methyl substituents appear to play a minor role in AChE inhibition. However, also in this case, it can be seen that the methyl derivatives were more potent AChE inhibitors than the ethyl derivatives. Thus, the strongest inhibitors were derivatives containing isopropylamino substituents (III) and (I), then dimethylamino substituents (II) and (IV), methyl substituents (II) and (IV) and the weakest ones were ethyl (I) and (III) derivatives, whereas inhibition potency and affinity strength values showed that aminoalkanol derivatives (I), (II), (III) and (IV) are slightly more potent inhibitors than Rivastigmine, which inhibits AChE from occurring in the cortex.

## 4. Materials and Methods

### 4.1. Reagents and Chemicals

Acetylcholinesterase human (EC. 3.1.1.7, AChE), Acetylthiocholine iodide (ACth), 5,5′-Dithiobis (2-nitrobenzoic) acid (DTNB), 4,4-dithio-bis-acid nitrobenzoic (DBAN), 5-thio-2-nitrobenzoic acid (NTB) were obtained from Sigma-Aldrich (Poznań, Poland). We used phosphate buffer (obtained from Beckman-Coulter, Brea, CA, USA). The compounds (I), (II), (III) and (IV) were synthesized and chemically analyzed (mass spectrometry and nuclear magnetic resonance spectroscopy) as described previously [37].

### 4.2. Instrumentation

A Beckman Coulter P/ACE MDQ CE system was used for electrophoretic analysis. The instrument had an autosampler along with a UV–Visible detector. All CE parameters were controlled using Karat software (v. 32). Separation was carried out using eCAP fused-silica capillary (total length: 30 cm, effective length: 20 cm, inner diameter: 50 μm, outer diameter: 375 μm).

### 4.3. Capillary Electrophoresis (CE) Conditions

Electrophoretic separations of 4,4-dithio-bis-acid nitrobenzoic (DBAN) (main enzyme reaction product) in the presence of acetylthiocholine (ACth), 5-thio-2-nitrobenzoic acid (NTB), 5,5′-dithio-bis-2-nitrobenzoic acid (DTNB), acetylcholinesterase (AChE) and inhibitors (I), (II), (III) and (IV) of acetylcholinesterase, and compounds (I), (II), (III) and (IV) by CE was performed using 100 mM phosphate buffer as a background electrolyte (BGE), at pH 7.5 adjusted with 100 mM sodium hydroxide. The samples were injected under 5 psi pressure for 3 s. The experiments were performed under constant current conditions (85 μA). The separation temperature was 20 °C, and the detection wavelength was set at 412 nm. Before the assay, the capillary was conditioned with 0.1 M NaOH for 10 min at 10 psi and with H_2_O for 10 min at 10 psi. Then, it was electro conditioned with the running buffer for 20 min at +10 kV and pressure-conditioned with the running buffer for 10 min at 10 psi. Before each injection, the capillary was washed with 1 M NaOH for 2 min, then with H_2_O for 2 min, and finally with BGE for 2 min. At the end of each working day, the capillary was rinsed for 10 min with 0.1 M NaOH, 10 min with BGE, and 10 min with deionized water at 15 psi. Then, the capillary was exposed to air for 10 min at 20 psi and left empty.

Sample mixtures containing 4,4-dithio-bis-acid nitrobenzoic (DBAN) (main enzyme reaction product) in the presence of acetylthiocholine (ACth), 5-thio-2-nitrobenzoic acid (NTB), 5,5′-dithio-bis-2-nitrobenzoic acid (DTNB), acetylcholinesterase (AChE) and inhibitors (I), (II), (III) and (IV) were introduced automatically by pressure injection (with 2 psi pressure for 5 s on the sample solution). At the day’s end, vials with anode and cathode buffer were emptied and running buffer was filled again at the start of the next day before the analysis. The detector wavelength was fixed at 412 nm. The appropriate parameters for analyzing DBAN were as follows: phosphate buffer (pH 7.5) of 100 mM concentration, 20 °C temperature, and 15 kV voltage.

### 4.4. Preparation of Stock and Working Standards

Primary stock standard solutions were prepared for compounds (I), (II), (III) and (IV) in deionized water (concentration of each = 50 mM). Primary stock standard solutions were also prepared for ACth separately using deionized water, with 332.5 mg of ACth dissolved in 25 mL of phosphate buffer (100 mM, pH 7.5), achieving 46.00 mM concentration. The prepared ACth solution was diluted with phosphate buffer (100 mM), and mixed working standard solutions were obtained (concentrations: 34.50, 23.00, 11.50, 5.75, 2.88, 1.44, 0.72, and 0.36 mM).

### 4.5. Sample Preparation

The AChE was dissolved in the 100 mM phosphate buffer, pH 7.5 and divided into several portions, which were stored at −20 °C. One aliquot was thawed each day and the AChE activity was checked. The working solutions of DNTB and ACth in appropriate concentrations were prepared immediately before use by dilution with the phosphate buffer.

Acetylcholinesterase activity was determined according to the method by Ellman et al. [47] and according to the procedure described by Rosilene R et al. [48] but slightly modified. Briefly: In nine test tubes, 1.7 mL phosphate buffer (100 mM concentration, pH 7.5) containing ACth (concentrations of 0.36–46.00 mM), 0.02 mL of (I), (II), (III) or (IV) inhibitor solution, 0.1 mL 4 mM 5,5′-dithio-bis-2-nitrobenzoic acid (DTNB) and 0.08 mL H_2_O was added. To another tube (zero sample, tube 10), 1.8 mL of phosphate buffer (100 mM concentration, pH 7.5) containing ACth (0.36 mM concentration), 0.1 mL 4 mM DTNB, 0.02 mL of (I), (II), (III) or (IV) inhibitor solution and 0.08 mL H_2_O was added. The tubes containing solutions were preincubated for 2 min at 25 °C. Then, 0.1 mL 50 mM AChE was introduced into tubes 1–9, at an interval of 1 min. All 10 samples were incubated for 3 min at 25 °C. After incubation, the analytical samples were introduced into the capillary. The color intensity of the obtained product was assessed at 412 nm. The results obtained from determining the inhibition type using the following equation are shown on the Lineweaver–Burk coordinate system in Figure 3, Table 1:

$$\frac{1}{V}=\frac{Km}{V\max[S]}+\frac{1}{V\max}$$

**Figure 8:**
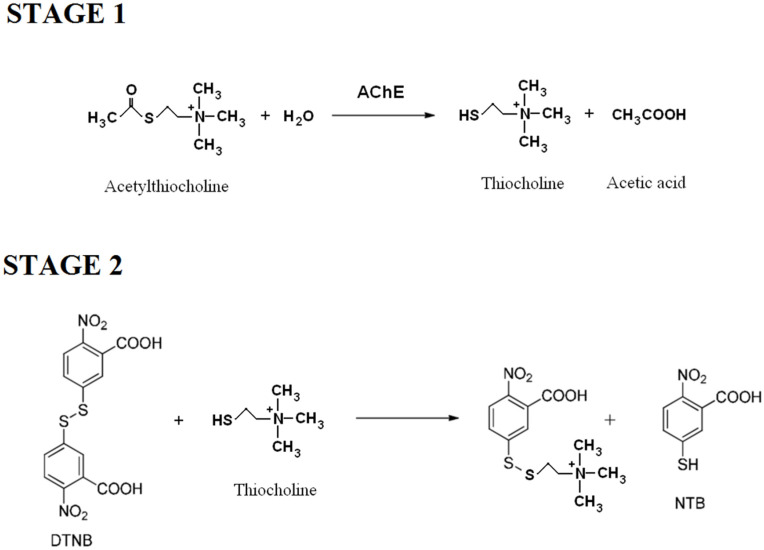
Reaction scheme for the determination of acetylcholinesterase activity.

Determination of inhibition effect of the compounds (I), (II) and (III), (IV) on AChE, was determined graphically as % activity depending on the changes in the concentrations of inhibitors (I) and (II). The IC_50_ values were obtained from activity (%) versus compounds (I), (II) and (III), (IV) plots. The inhibition constants (Ki) were calculated from the plotted Lineweaver–Burk curves for the four concentrations of the inhibitors (I), (II), (III) and (IV). The method was based on two steps (Figure 8).

Free Thiocholine (stage 1), formed from Acetylthiocholine under the influence of AChE, reacts then with DTNB releasing a certain amount of NTB (stage 2), the concentration of which depends on the AChE activity.

#### Docking Studies

To assess possible ligand–AChE interaction, molecular docking experiments were performed using the PyRx docking tool [49] via the Autodock VINA software [50,51]. The three-dimensional (3D) crystal structures of human AChE enzyme was obtained from the RCSB Protein Data Bank. For docking studies two crystal structures 6o4w and 6o4x were selected. The crystallographic structure was resolved with a resolution of 2.35 and 2.30 in complex with the inhibitors of the Acetylcholinesterase donepezil and 9 aminoacridine respectively.

The three-dimensional (3D) structures (PDB format) of both AChE structures were analyzed and prepared for docking studies using PyMOL software (The PyMOL Molecular Graphics System, Version 2.0 Schrödinger, LLC). The binding pocket was selected on the basis of the literature data and consists of both the active site and the peripheral anionic site (PAS). Both sites were located on the basis of the presence of amino acids classified as active sites (SER 203, HIS 4447, GLU 334, TRP 86) and PAS (TYR 70, TYR 14, TYR 341, TRP 286).

The PDB AChE files (6o4w, 6o4x) have been uploaded to the PyRx utility linked to the Autodock VINA. The macromolecule was converted to the pdbqt format, which automatically removes solvent particles, adds hydrogen and performs Gasteiger charges calculations. The center of the mesh was placed in the active sites based on the position of selected amino acids classified as a binding site pocket (SER 203, HIS 4447, GLU 334, TRP 86, TYR 70, TYR 14, TYR 341, TRP 286). The dimension of grid box 28 × 26 × 25 was set to cover both sites in crystal structures.

As ligands all four structures (I, II, III, IV) were selected, as well as the structure of donepezil, as a model inhibitor of AChE. No constraints were used in the docking process. The exhaustive value 40 was used to maximize the conformational binding analysis. The generated docked complexes were selected based on binding affinity values (kcal/mol) and binding interaction patterns (hydrogen, hydrophobicity, and electrostatic), which were analyzed for higher scored conformation. Graphical representations of all docked complexes were made using the Discovery studio visualizer version 4.0 (BIOVIA, San Diego, CA, USA).

## 5. Conclusions

As aminoalkanol derivatives were not recognized as PAP inhibitors and not reported in the literature thus far, we attempted to analyze their effect on the activity of AChE. Our study showed that AChE inhibition can be assessed simply with an in vitro drug metabolic system using CE analysis.

All four aminoalkanol derivatives showed a competitive type of inhibition of AChE. The Km, Vmax, line angles of Lineweaver–Burk, Ki and IC_50_ showed that derivative (III) was the most potent AChE inhibitor. Derivatives (IV) and (I) showed less inhibition. Derivative (II) was the weakest AChE inhibitor of all four derivatives. The obtained results suggest that the inhibition potency depends on the type of substituent present in the main structure of the compound. Isopropylamine and dimethylamine substituents exert a stronger AChE inhibitory effect than ethyl and methyl substituents. The differences in the IC_50_ values are slight and, to simplify considerably, one could say that with such minimal differences all the studied compounds inhibit acetylcholinesterase with the same IC_50_. However, the CE method we developed allows us to capture such minimal differences.

Additionally, docking studies suggest that studied compounds bind with the PAS and do not enter into the catalytic pocket due to the presence of the sterically extended substituent.

## 6. Future Perspectives

The promising results of the research presented in this paper encourage us to continue research on the search for new derivatives with a much greater effectiveness of inhibiting acetylcholinesterase. Therefore, in the longer term, our target is to introduce more elaborate or completely different types of substituents (functional groups) into the main structure of the molecule. Such a solution will allow the creation of a large series of new derivatives with different potency. From among these derivatives, we want to obtain (isolate) compounds with the highest activity, the potency of acetylcholinesterase inhibition and the ones with the lowest toxicity.

## Figures and Tables

**Table 1:** Regression equation and quantification for acetylthiocholine for 6 replicates for each sample (*n* = 6) in the concentration range 0.36–46.00 mM, in the presence of inhibitors (**I**), (**II**), (**III**) and (**IV**) at a concentration of 0.08 mM.

Concentration Inhibitors (I), (II), (III) and (IV) (mM)	Linearity Range of Substrate (Acetylthiocholine) (mM)	R^2^	RSD (%)	LOD (mM)	LOQ (mM)	Regression Equation	Standard Deviation	
							Slope	Intercept
0	0.36–46.00	0.998	2.53	0.10	0.36	y = 1.546x + 1.544	±0.039	±0.025
(**I**) 0.08	0.36–46.00	0.997	3.21	0.12	0.36	y = 2.785x + 1.543	±0.065	±0.041
(**II**) 0.08	0.36–46.00	0.998	2.98	0.15	0.36	y = 2.736x + 1.542	±0.061	±0.036
(**III**) 0.08	0.36–46.00	0.998	3.35	0.13	0.36	y = 2.989x + 1.580	±0.051	±0.040
(**IV**) 0.08	0.36–46.00	0.998	3.42	0.15	0.36	y = 2.808x + 1.559	±0.055	±0.043

LOD, limit of detection; LOQ, limit of quantification; RSD, relative standard deviation.

**Table 2:** The analytical data describing effects of acetylcholinesterase inhibition by aminoalkanol derivatives (**I**), (**II**), (**III**) and (**IV**) and regression equation for acetylthiocholine for 6 replicates for each sample (*n* = 6) at the concentration range 0.36–46.00 mM, in the presence of inhibitors (**I**), (**II**), (**III**) and (**IV**), at a concentration of 0.01, 0.02, 0.04, 0.06 and 0.08 mM.

Compound (I)				
Concentration (mM)	Straight Line Equation	R^2^	Tilt Angle (°)	
(**I**) 0.00	y = 1.5467x + 1.5447	0.9989 ± 0.0009	57.12 ± 0.05	
(**I**) 0.01	y = 1.6989x + 1.5868	0.9984 ± 0.0008	59.52 ± 0.04	
(**I**) 0.02	y = 1.8424x + 1.6078	0.9981 ± 0.0015	69.90 ± 0.06	
(**I**) 0.04	y = 2.2472x + 1.5579	0.9992 ± 0.0007	65.42 ± 0.05	
(**I**) 0.06	y = 2.6975x + 1.5421	0.9986 ± 0.0012	69.66 ± 0.05	
(**I**) 0.08	y = 2.7965x + 1.5284	0.9988 ± 0.0010	70.32 ± 0.03	
**Concentration (mM)**	**Km (mM/mL)**	**Vmax (mM/min)**	**Ki (mM)**	**IC_50_ (mM)**
(**I**) 0.00	1.00 ± 0.02	0.65 ± 0.02	---	---
(**I**) 0.01	1.07 ± 0.01	0.63 ± 0.02	14.31 ± 0.02	
(**I**) 0.02	1.15 ± 0.03	0.62 ± 0.03	11.90 ± 0.04	
(**I**) 0.04	1.44 ± 0.02	0.64 ± 0.01	2.87 ± 0.04	0.037 ± 0.001
(**I**) 0.06	1.75 ± 0.02	0.65 ± 0.01	1.64 ± 0.03	
(**I**) 0.08	1.80 ± 0.01	0.65 ± 0.01	1.35 ± 0.03	
**Compound (II)**				
**Concentration (mM)**	**Straight Line Equation**	**R^2^**	**Tilt Angle (°)**	
(**II**) 0.00	y = 1.5467x + 1.5447	0.9989 ± 0.0009	57.12 ± 0.05	
(**II**) 0.01	y = 1.6950x + 1.6198	0.9986 ± 0.0012	59.46 ± 0.03	
(**II**) 0.02	y = 1.7820x + 1.5882	0.9976 ± 0.0018	59.60 ± 0.08	
(**II**) 0.04	y = 2.1631x + 1.5495	0.9981 ± 0.0016	65.19 ± 0.06	
(**II**) 0.06	y = 2.6080x + 1.5316	0.9991 ± 0.0008	69.02 ± 0.05	
(**II**) 0.08	y = 2.7504x + 1.5348	0.9988 ± 0.0009	70.02 ± 0.04	
**Concentration** **(mM)**	**Km (mM/mL)**	**Vmax (mM/min)**	**Ki (mM)**	**IC_50_ (mM)**
(**II**) 0.00	1.00 ± 0.02	0.65 ± 0.02	---	---
(**II**) 0.01	1.05 ± 0.02	0.62 ± 0.03	22.24 ± 0.03	
(**II**) 0.02	1.09 ± 0.03	0.63 ± 0.02	14.01 ± 0.03	
(**II**) 0.04	1.40 ± 0.02	0.65 ± 0.01	2.98 ± 0.05	0.039 ± 0.001
(**II**) 0.06	1.70 ± 0.02	0.65 ± 0.01	1.83 ± 0.03	
(**II**) 0.08	1.79 ± 0.02	0.65 ± 0.01	1.42 ± 0.02	
**Compound (III)**				
**Concentration (mM)**	**Straight Line Equation**	**R^2^**	**Tilt Angle (°)**	
(**III**) 0.00	y = 1.5467x + 1.5447	0.9989 ± 0.0009	57.12 ± 0.05	
(**III**) 0.01	y = 1.7325x + 1.5761	0.9988 ± 0.0007	60.01 ± 0.03	
(**III**) 0.02	y = 1.9031x + 1.5936	0.9978 ± 0.0010	62.28 ± 0.03	
(**III**) 0.04	y = 2.3998x + 1.5596	0.9986 ± 0.0006	67.38 ± 0.04	
(**III**) 0.06	y = 2.8117x + 1.5726	0.9995 ± 0.0004	70.42 ± 0.02	
(**III**) 0.08	y = 2.9892x + 1.5806	0.9989 ± 0.0009	71.50 ± 0.03	
**Concentration (mM)**	**Km (mM/mL)**	**Vmax (mM/min)**	**Ki (mM)**	**IC_50_ (mM)**
(**III**) 0.00	1.00 ± 0.02	0.65 ± 0.02	---	---
(**III**) 0.01	1.09 ± 0.01	0.63 ± 0.01	10.20 ± 0.01	
(**III**) 0.02	1.19 ± 0.03	0.63 ± 0.02	5.19 ± 0.02	
(**III**) 0.04	1.50 ± 0.01	0.64 ± 0.03	2.01 ± 0.04	0.033 ± 0.001
(**III**) 0.06	1.80 ± 0.01	0.64 ± 0.03	1.25 ± 0.03	
(**III**) 0.08	1.90 ± 0.01	0.63 ± 0.02	1.11 ± 0.02	
**Compound (IV)**				
**Concentration (mM)**	**Straight Line Equation**	**R^2^**	**Tilt angle (°)**	
(**IV**) 0.00	y = 1.5467x + 1.5447	0.9989 ± 0.0009	57.12 ± 0.05	
(**IV**) 0.01	y = 1.7032x + 1.5791	0.9987 ± 0.0010	59.58 ± 0.04	
(**IV**) 0.02	y = 1.7387x + 1.5842	0.9997 ± 0.0002	60.09 ± 0.04	
(**IV**) 0.04	y = 2.2025x + 1.5617	0.9989 ± 0.0009	65.58 ± 0.03	
(**IV**) 0.06	y = 2.7115x + 1.5761	0.9988 ± 0.0009	69.76 ± 0.03	
(**IV**) 0.08	y = 2.8082x + 1.5592	0.9989 ± 0.0009	70.40 ± 0.03	
**Concentration (mM)**	**Km (mM/mL)**	**Vmax (mM/min)**	**Ki (mM)**	**IC_50_ (mM)**
(**IV**) 0.00	1.00 ± 0.02	0.65 ± 0.02	---	---
(**IV**) 0.01	1.08 ± 0.03	0.63 ± 0.02	13.10 ± 0.01	
(**IV**) 0.02	1.10 ± 0.03	0.63 ± 0.02	10.33 ± 0.02	
(**IV**) 0.04	1.41 ± 0.03	0.64 ± 0.03	2.45 ± 0.03	0.035 ± 0.001
(**IV**) 0.06	1.72 ± 0.01	0.63 ± 0.01	1.39 ± 0.03	
(**IV**) 0.08	1.83 ± 0.03	0.64 ± 0.03	1.22 ± 0.02	

**Table 3:** Effect of the type of substituents occurring in the main structure of aminoalkanol derivatives (**I**), (**II**), (**III**) and (**IV**) on inhibitory potency (IC_50_) and affinity strength (Ki) of AChE.

Compound	R_1_	R_2_	R_3_	Ki [mM]	IC_50_ [mM]	Docking Energy [kcal/mol]
Derivative (**I**)	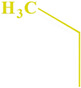	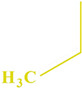	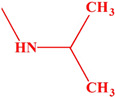	1.35	0.037	−8.1
Derivative (**II**)	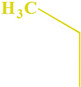	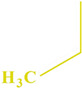	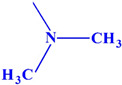	1.42	0.039	−8.5
Derivative (**III**)	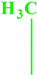	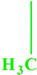	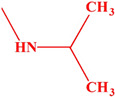	1.11	0.033	−8.9
Derivative (**IV**)	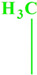	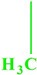	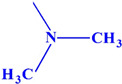	1.22	0.035	−9.2

## Data Availability

Not applicable.

## Supplementary Materials

The following supporting information can be downloaded at: https://www.mdpi.com/article/10.3390/ijms23010270/s1.
