# Supplementary material for: In Vitro and In Silico Kinetic Studies of Patented 1,7-diEthyl and 1,7-diMethyl Aminoalkanol Derivatives as New Inhibitors of Acetylcholinesterase
Source: Int J Mol Sci. 2021 Dec 27;23(1):270. doi: 10.3390/ijms23010270 (PMC8745342; doi:10.3390/ijms23010270)
Supplement: Supplementary file 1 [file ijms-23-00270-s001.zip › ijms-1501862-supplementary.pdf]

Table S1. Competitive inhibition. Values characterizing the course of the reaction in the presence of the inhibitor (I), (II), (III) and (IV) of acetylcholinesterase. The data present the changes of peak area corresponding to the increase in the amount of reaction product (4,4-dithio-bis-acid nitrobenzoic) and reaction speed (V_0 and $1/V_0$) in the system without inhibitors ($C_{0.00}$) and with inhibitors (I), (II), (III) and (IV) at concentrations of 0.08 mM ($C_{0.08}$) and the other concentration from 0.00 mM to 0.08 mM . The changes in enzymatic reaction parameters were registered in the presence of the substrate (acetylthiocholine) at specific concentrations in the range of 0.36–46.00 mM.

Substrate concentration (acetylthiocholine) [S] (mM)	$\frac{1}{[S]}$									
					Peak area [PA]					
				Speed reaction = amount of the produced product/min						
					Amount of the product (4,4-dithio-bis-acid nitrobenzoic) (mM/min)					
			**$C_{0.00}$**			**$C_{0.08}$ (I)**			**$C_{0.08}$ (II)**	
		Mean	SD	%RSD	Mean	SD	%RSD	Mean	SD	%RSD
0.36	2.78	63196	1453.5	2.3	49766	1343.7	2.7	56086	1738.7	3.1
0.72	1.39	115890	2433.7	2.1	91263	2372.8	2.6	102853	2879.9	2.8
1.44	0.69	163569	3925.7	2.4	128810	3220.3	2.5	145167	3919.5	2.7
2.88	0.35	217345	4129.6	1.9	171159	3765.5	2.2	192894	4822.4	2.5
5.75	0.17	263046	5260.9	2.0	207149	4350.1	2.1	233453	5369.4	2.3
11.50	0.09	293673	5579.8	1.9	231267	4625.3	2.0	260635	5733.9	2.2
23.00	0.04	314657	5349.2	1.7	247792	4708.0	1.9	279258	5585.2	2.0
34.50	0.03	318064	5089.0	1.6	250475	4508.6	1.8	282281	5363.3	1.9
46.00	0.02	319546	5112.7	1.6	251643	4529.6	1.8	283597	4821.1	1.7
			**$C_{0.08}$ (III)**			**$C_{0.08}$ (IV)**				
		Mean	SD	%RSD	Mean	SD	%RSD			
		39497	789.9	2.0	45817	1145.4	2.5			
		72431	1376.2	1.9	84020	2016.5	2.4			
		102230	1942.4	1.9	118587	2846.1	2.4			
		135840	2445.1	1.8	157575	3466.7	2.2			
		164404	2794.9	1.7	190708	4004.9	2.1			
		183545	2936.7	1.6	212913	4258.3	2.0			
		196660	3146.6	1.6	228126	4106.3	1.8			
		198790	3180.6	1.6	230596	4150.7	1.8			
		199716	2995.7	1.5	231671	4170.1	1.8			
					V_0 (mM/min)					
			**$C_{0.00}$**			**$C_{0.08}$ (I)**			**$C_{0.08}$ (II)**	
		Mean	SD	%RSD	Mean	SD	%RSD	Mean	SD	%RSD
		0.17	0.002	1.22	0.11	0.001	1.35	0.11	0.001	1.45
		0.27	0.004	1.54	0.18	0.003	1.62	0.19	0.002	1.82
		0.38	0.007	1.77	0.28	0.005	1.85	0.29	0.004	1.93
		0.50	0.012	2.47	0.43	0.008	1.98	0.43	0.008	2.15
		0.53	0.015	2.76	0.49	0.013	2.75	0.49	0.011	2.86
		0.59	0.020	3.37	0.56	0.019	3.42	0.56	0.016	3.11
		0.61	0.021	3.44	0.59	0.021	3.59	0.59	0.020	3.58
		0.63	0.018	3.87	0.65	0.024	3.74	0.65	0.022	3,90
		0.67	0.026	3.92	0.67	0.027	4.12	0.67	0.024	4,41
			**$C_{0.08}$ (III)**			**$C_{0.08}$ (IV)**				
		Mean	SD	%RSD	Mean	SD	%RSD			
		0.10	0.001	1.12	0.10	0.002	2.15			
		0.18	0.002	1.18	0.19	0.004	2.33			
		0.28	0.005	1.65	0.29	0.008	2.85			
		0.42	0.010	2.40	0.43	0.013	3.04			
		0.49	0.013	2.72	0.49	0.017	3.42			

0.56	0.019	3.45	0.56	0.022	3.89
0.59	0.022	3.78	0.59	0.024	4.05
0.65	0.025	3.85	0.65	0.028	4.28
0.64	0.024	3.80	0.65	0.030	4.67

$\frac{1}{Vo}$ (min/mM)

**$C_{0.00}$**			**$C_{0.08}$ (I)**			**$C_{0.08}$ (II)**		
Mean	SD	%RSD	Mean	SD	%RSD	Mean	SD	%RSD
5.85	0.070	1.20	9.30	0.104	1.12	9.19	0.104	1.13
3.70	0.056	1.52	5.46	0.064	1.18	5.40	0.064	1.19
2.60	0.046	1.78	3.55	0.058	1.65	3.50	0.057	1.64
2.00	0.049	2.46	2.30	0.055	2.40	2.30	0.054	2.38
1.90	0.052	2.77	2.05	0.056	2.72	2.05	0.055	2.73
1.70	0.057	3.38	1.80	0.062	3.45	1.80	0.062	3.46
1.65	0.056	3.42	1.70	0.064	3.78	1.70	0.064	3.77
1.60	0.062	3.89	1.55	0.059	3.85	1.55	0.059	3.82
1.50	0.058	3.92	1.50	0.049	3.27	1.50	0.059	3.91

**$C_{0.08}$ (III)**			**$C_{0.08}$ (IV)**		
Mean	SD	%RSD	Mean	SD	%RSD
10.1	0.093	0.93	9.85	0.110	1.12
5.70	0.071	1.26	5.40	0.064	1.18
3.55	0.066	1.86	3.50	0.058	1.65
2.40	0.066	2.75	2.30	0.055	2.40
2.05	0.060	2.94	2.05	0.056	2.72
1.80	0.064	3.58	1.80	0.062	3.45
1.70	0.063	3.75	1.70	0.064	3.78
1.55	0.044	2.87	1.55	0.059	3.85
1.50	0.050	3.36	1.50	0.054	3.62
